# [Retracted] MicroRNA‑675 inhibits cell proliferation and invasion in melanoma by directly targeting metadherin

**DOI:** 10.3892/mmr.2023.12986

**Published:** 2023-03-27

**Authors:** Ke Liu, Junjun Jin, Kunjie Rong, Lukai Zhuo, Pingsong Li

Mol Med Rep 17: 3372–3379, 2018; DOI: 10.3892/mmr.2017.8264

Following the publication of this paper, it was drawn to the Editor's attention by a concerned reader that the western blotting data shown in Fig. 5A and the cell migration and invasion assay data shown in Fig. 5C were strikingly similar to data appearing in different form in other articles by different authors at different research institutes, several of which have been retracted. Owing to the fact that the contentious data in the above article were already under consideration for publication, or had already been published, prior to its submission to *Molecular Medicine Reports*, the Editor has decided that this paper should be retracted from the Journal. After having been in contact with the authors, they agreed with the decision to retract the paper. The Editor apologizes to the readership for any inconvenience caused.

